# High normal values of circulating immune cell subsets before surgery may be protective against development of postoperative acute kidney injury

**DOI:** 10.1186/2197-425X-3-S1-A626

**Published:** 2015-10-01

**Authors:** K Ehehalt, P Renner, F Zeman, K Pfister, P Riquelme, BM Graf, EK Geissler, P Kasprzak, HJ Schlitt, T Bein, JA Hutchinson, I Gocze

**Affiliations:** Dep. of Anesthesia, University Medical Center Regensburg, Regensburg, Germany; Dep. of Surgery and Experimental Surgery, University Medical Center Regensburg, Regensburg, Germany; Center for Clinical Studies, University Medical Center Regensburg, Regensburg, Germany

## Introduction

Occlusion of renal artery during stenting procedure by endovascular aortic repair leads to ischemic-reperfusion injury (IRI). Additionally, significant amount of intravenous contrast agent is used during the procedure. Therefore acute kidney injury (AKI) is one of the most common postoperative complications. Current experimental data suggest an important role of systemic immune reaction in development of AKI after IRI and nephrotoxic agents. However, robust analysis of cellular immune response in clinical setting and its relation to AKI after IRI and iv contrast have not been previously reported.

## Objectives

We evaluated the perioperative risk factors for AKI after elective aortic surgery. Moreover, whole blood immune phenotype monitoring (leukocyte, T cells, B cell and dendritic cell subsets) was used for assessing the systemic immune response within first 24 hours after surgery in all patients, in patients with elevated cell cycle arrest markers and in patients with AKI (according to KDIGO 2012).

## Methods

In this prospective study, with 18 consecutively included patients, the perioperative risk factors were evaluated. In addition, laboratory parameters such as creatinine, interleukin 6 (IL-6), central venous oxygen saturation, lactate, hemoglobin, creatinine kinase and urinary cell cycle arrest biomarkers TIMP-2 and IGFBP7 (tissue inhibitor of metalloproteinase 2 and insulin-like growth factor-binding protein 7) were measured before surgery, immediately after surgery and 24 hours after start of surgery. Finally a standardized flow cytometry immune monitoring was used for assessment of circulating absolute cell numbers before and 24 hours after start of surgery.

## Results

In the logistic regression, age, duration of surgery, IL-6 after surgery, [TIMP-2].[IGFBP7] after surgery and at 24 hours and creatinine at 24 hours were associated with development of AKI. All patients showed strong inflammatory response with drop in absolute numbers of circulating cells subsets within first 24 hours as a consequence of cells migration from blood compartment in to the organs (in 59 of 62 measured subsets: CD3, CD4 p < 0.001, CD8, CD4CD8 p = 0.001). Patients with tubular cell cycle arrest had higher decrease of circulating cells compared to patients without elevation of [TIMP-2].[IGFBP7]. Patients who developed AKI showed less pronounced changes in circulating cells after surgery (CD3 p = 0.09, CD4 p = 0.07, CD8 p= 0.17, CD4CD8 p = 0.36), which was due to significantly lower absolute numbers of cellular subsets before surgery.

## Conclusions

Patients with strong inflammatory response after surgery are at risk for tubular cellular arrest. Consecutive AKI developed predominately patients with low normal values of immune cells before surgery. Low normal values of cell subsets may be the consequence of undiagnosed subclinical disease, which may increase the risk of postoperative AKI.

(KISMED Study, NCT01915446).

